# Study of Testicular Structure in Fetuses with Prune Belly Syndrome

**DOI:** 10.1155/2017/3254980

**Published:** 2017-05-21

**Authors:** Luciano A. Favorito, Suelen F. Costa, Waldemar S. Costa, Rodrigo Vieiralves, Fabio O. Bernardo, Francisco J. B. Sampaio

**Affiliations:** Urogenital Research Unit, State University of Rio de Janeiro, Rio de Janeiro, RJ, Brazil

## Abstract

**Purpose:**

To compare the structure of the testis in fetuses with prune belly syndrome (PBS) to normal controls.

**Materials and Methods:**

We studied 6 testes obtained from 3 fetuses with PBS and 14 testes from 7 male fetuses. The testicular specimens were cut into 5-*μ*m thick sections and stained with hematoxylin and eosin (HE), to observe the seminiferous tubules; Weigert's solution to observe elastic fibers; and picrosirius red to observe collagen. The images were captured with an Olympus BX51 microscope and Olympus DP70 camera. The stereological analysis was done with the Image Pro and Image J programs. Means were statistically compared using the Mann–Whitney *U* test (*p* < 0.005).

**Results:**

Quantitative analysis documented no differences (*p* = 0.4) in number of seminiferous tubules (ST) in PBS testes (mean = 8.87%, SD = 1.59), when compared to the control (mean = 11.4%, SD = 2.99) and no differences (*p* = 0.8) in diameter of ST in PBS testes (mean = 52.85 *μ*m, SD = 1.58) when compared to the control group (mean = 53.17 *μ*m, SD = 1.55), but we did observe a lower number (*p* = 0.0002) of Leydig cells in the PBS testes (mean = 67.03% and SD = 3.697) when compared to the control group (mean = 90.1% and SD = 2.986).

**Conclusions:**

Our study showed a lower concentration of Leydig cells in the triad syndrome fetuses.

## 1. Introduction

Prune belly syndrome (PBS) or triad syndrome is a disorder characterized by deficiency or hypoplasia of the abdominal muscles, malformations of the urinary tract such as large and hypotonic bladders, dilated and tortuous ureters, and bilateral cryptorchidism [1]. Intra-abdominal cryptorchidism and infertility are universal features of prune belly syndrome [2]. The cause of cryptorchidism in this syndrome is unknown, but it is speculated that anatomical changes in the anterior abdominal wall hinder the increase of intra-abdominal pressure, one of the factors necessary for testicular descent [3]. Recently, important alterations in gubernaculum testis structure were demonstrated in fetuses with PBS [4].

Previous studies have shown that the germinative epithelium is significantly reduced and that seminiferous tubule diameter increases with age in sufferers of prune belly syndrome [5–8]. Nevertheless, structural and quantitative analyses of seminiferous tubules, extracellular matrix, and interstitial cells during the human fetal period in PBS are scarce in the literature.

The hypothesis stated in our study is as follows: Are the seminiferous tubule structure and Leydig cell number similar in PBS and normal fetuses? Does the increased intra-abdominal pressure in PBS cause alterations in testis structure?

Therefore, the objective of this paper is to compare and contrast the structure of the testis in fetuses with prune belly syndrome (PBS) to normal controls.

## 2. Material and Methods

The experimental protocol described here was approved by the ethics committee on human experimentation of our university. This study was carried out in accordance with the ethical standards of the hospital's institutional committee on human experimentation.

We studied 6 testes obtained from 3 male fetuses with PBS (with typical aspect of the anterior abdominal wall, obstruction in prostatic urethra, enlarged bladders, and bilateral hydronephrosis) and 14 testes obtained from 7 male fetuses without anomalies. The fetuses were macroscopically well preserved. The gestational age of the fetuses was determined in weeks postconception (WPC), according to the foot-length criterion, which is currently considered the most acceptable parameter to calculate gestational age [9–11]. The fetuses were also evaluated regarding crown-rump length (CRL) and body weight immediately before dissection. The same observer analyzed the measurements.

After the measurements, the fetuses were carefully dissected with the aid of a stereoscopic lens with 16/25x magnification. The abdomen and pelvis were opened to identify and expose the urogenital organs and inguinal canal and to show the testes' position.

Testicular position was classified after dissection into (a) abdominal, when the testis was proximal to the internal ring; (b) inguinal, when the testis was found between the internal and external inguinal rings; and (c) scrotal, when the testis had passed beyond the external inguinal ring and was inside the scrotum.

Each testis was separated from the other structures and fixed in 10% buffered formalin and routinely processed for paraffin embedding, after which 5-*μ*m thick sections were obtained at 200-*μ*m intervals. Sections were stained with hematoxylin-eosin to assess the integrity of the tissue and the structure of seminiferous tubules. The following staining methods were used: Weigert's resorcin fuchsin with previous oxidation to observe elastic system fibers and picrosirius red with polarization for observation of different collagen types.

**Figure 1:**
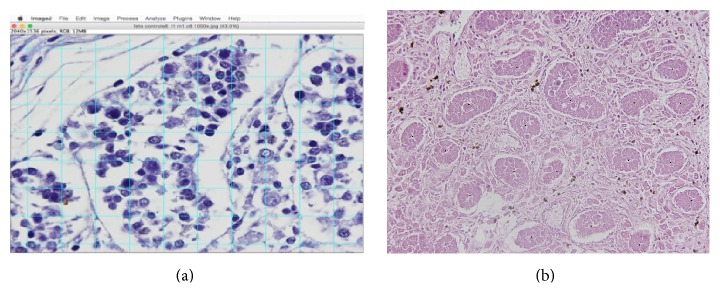
(a) Morphometric analysis of the seminiferous tubules and Leydig cells in fetal testis. The diameter of seminiferous tubules was measured using the program at least five times for each specimen and was determined by the contour of the epithelium. HE ×400. (b) Morphometric analysis of the seminiferous tubules and Leydig cells in fetal testis. To quantify the number of seminiferous tubules we used the Image J Test grid software to determine the volumetric density (*Vv*). HE ×400.

The seminiferous tubules and Leydig cells were quantified by a stereological method [12–14]. We studied 5 microscopic fields chosen at random, totaling 25 test areas studied for each testis for the quantitative analysis. We used the Image J software, version 1.46r, loaded with its own plug-in (http://rsb.info.nih.gov/ij/). All sections were photographed with a digital camera (DP70, Olympus America, Inc., Melville, New York) under the same conditions at a resolution of 2,040 pixels, directly coupled to the microscope (BX51, Olympus America, Inc.) and stored in a TIFF file. The diameter of the seminiferous tubules was measured through the “straight” tool of the Image J software. This tool allows drawing a line on the tubule and calculating its diameter. Five tubules were analyzed in each microscopic field (Figure 1(a)).

To quantify the number of seminiferous tubules we used the Image J software to determine the volumetric density (*Vv*) of the tubules (Figure 1(b)). Results for each field were obtained through the quantification assessment method, to count all tubules of each section. The arithmetic mean of the quantification in 5 fields of each section was determined. Afterwards, we obtained the mean quantification value for the 5 sections studied from each testis (total of 25 test areas). To count interstitial cells, we used Image J software cell with the counter tool, selecting and marking all the cells present between the seminiferous tubules.

Means were statistically compared using the unpaired* t*-test and the Mann–Whitney *U* test for all categorical variables and Wilcoxon rank sum tests were used for continuous variables. All tests were two-sided and a *p* value < 0.05 was considered statistically significant.

## 3. Results

The prune belly fetuses ranged in age from 17 to 31 WPC, weighed between 240 and 2150 g, and had crown-rump length between 18 and 43 cm. The fetuses in the control group ranged in age from 12 to 35 WPC, weighed between 210 and 2860 g, and had crown-rump length between 18 and 34 cm (Table 1). The 6 testes in fetuses with prune belly syndrome and the 14 testes in the control group had abdominal testis. We did not observe cases of epididymal anomalies in our sample.

**Figure 2:**
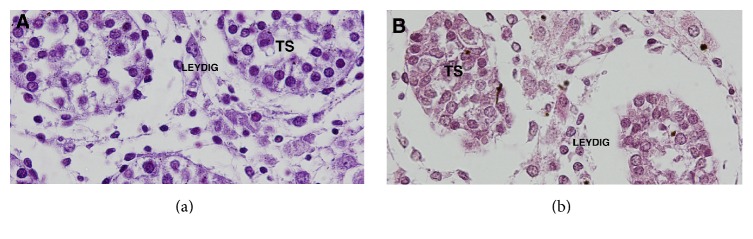
Analysis of testicular structure. (a) Photomicrograph showing the seminiferous tubules (TS) and Leydig cells arrangement in the control group. Fetus with 21 WPC. HE ×400. (b) Photomicrograph showing the seminiferous tubules (TS) and Leydig cells arrangement in prune belly group. Fetus with 31 WPC. HE ×400.

Quantitative analysis documented no differences (*p* = 0.4) in number of seminiferous tubules in PBS testes (mean = 8.87%, SD = 1.59, and SE = 0.9179), when compared to the control group (mean = 11.4%, SD = 2.99, and SE = 1.499).  Figure 2 shows the seminiferous tubules' arrangement in normal and prune belly testes.

Quantitative analysis documented no differences (*p* = 0.8) in diameter of seminiferous tubules in PBS testes (mean = 52.85 *μ*m, SD = 1.58, and SE = 0.9142), when compared to the control group (mean = 53.17 *μ*m, SD = 1.55, and SE = 4.717).

Quantitative analysis documented a significantly smaller number (*p* = 0.0002) of Leydig cells in the PBS testes (mean = 67.03% and SD = 3.697), when compared to the control group (mean = 90.1% and SD = 2.986) (Figure 2).

**Figure 3:**
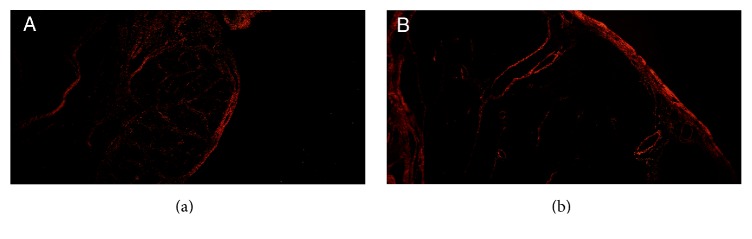
Analysis of collagen distribution. (a) The photomicrograph shows predominance of red in the testis of a control group fetus with 23 WPC, suggesting collagen type I presence. Picrosirius red with polarization ×20. (b) The photomicrograph shows predominance of red in prune belly fetal gubernaculum, suggesting collagen type I presence in a fetus with 31 WPC. Picrosirius red with polarization ×20.

We did not observe elastic fibers and smooth muscle cells in the seminiferous tubules in PBS and in the control group. Picrosirius red with polarization photomicrographs presented no differences in colors between the groups. The analysis showed predominance of red in both PBS and control fetal testes, suggesting collagen type I presence (Figure 3).

## 4. Discussion

Bilateral cryptorchidism is characteristic of prune belly syndrome [1–3]. The most important theories to explain bilateral cryptorchidism in this syndrome are (a) impaired contraction of the muscles of the abdominal wall; (b) mechanical obstruction due to bladder distention; (c) structural alteration of the inguinal canal, which hampers the passage of the testis; and (d) structural alterations in gubernaculum testis [1–4].

Patients with cryptorchidism have a higher risk of developing testicular cancer and infertility. Randomized volumetric and histological studies have shown that early treatment before the age of one year is beneficial for testicular development and future spermatogenesis compared to later treatment [15, 16]. Despite surgical treatment, impaired fertility (33% in unilateral cases and 66% in bilateral undescended testes) and a cancer risk 5–10 times greater than normal is observed over time [15]. In PBS, the germinative epithelium is significantly reduced so testis descent should be performed in the newborn period to enhance the chances of fertility [5–8, 17]. There are reports about live births of male infants resulting from intracytoplasmic sperm injection (ICSI) using spermatozoa from patients with PBS [18].

Studies of testicular interstitial cells and seminiferous tubules in fetuses and/or patients with PBS are scarce in the literature. In an elegant study, Orvis et al. [5] demonstrated that fetuses with PBS had marked Leydig cell hyperplasia in contrast to the other groups. In the present study, we did not observe significant alterations in Leydig cell volume, but the PBS testes had fewer interstitial cells than the control group, suggesting that testicular histological alterations in PBS begin during the fetal period. Choudhury et al. [8] studied the diameter of seminiferous tubules in human fetuses and in cadavers and observed that the diameter of the tubules increased with age. A previous study in human fetuses demonstrated that the diameter of seminiferous tubules increased significantly with fetal age [19].

Testicular histology was reviewed in boys with PBS and no spermatogonia was seen [6]. However, Uehling et al. [7] showed that all patients with PBS studied had atypical germ cells with large nuclei and prominent nucleoli, and intense alkaline phosphatase staining localized in the cytoplasmic membrane, suggesting developmental arrest in undescended testes associated with prune belly syndrome. Uehling et al. [7] also studied the germ cells but did not study the seminiferous tubule structure. Our study is the first to our knowledge to analyze the diameter and the number of seminiferous tubules in fetuses with PBS. Our findings showed no differences in tubular structure between the PBS and control groups. We did not analyze the germ cells, but the analysis of tubule structure is new and could be important to understand the structural alterations in PBS.

In an interesting study about apoptosis during the human fetal period, Helal et al. [20] found a decrease in the proportion of Sertoli cells with gestational age and no significant change in the germ cells and Leydig cells in relation to gestational age. However, O'Shaughnessy et al. [19] analyzed 57 human fetuses and demonstrated that Leydig cell number increased exponentially during the initial part of the second trimester, but by the end of the second trimester, the numbers were static.

In our sample we did not observe elastic fibers in any of the testes analyzed. This may indicate that this extracellular matrix component appears only in the third gestational trimester in the fetal testis. Previous studies have shown the existence of elastic system fibers in other human fetal genitourinary organs [21]. Collagen provides tensile strength, but overaccumulation can inhibit contractility and conduction of electrical impulses through the visceral wall [22, 23]. In our study, the qualitative analysis did not reveal differences in the distribution of collagen in the fetal testes between the groups.

Some limitations of this study should be mentioned: (a) unequal WPC distribution between PBS and control fetuses and (b) small sample size, since prune belly fetuses are rare. However, despite the sample size limitation, the observations still provide important new information.

## 5. Conclusions

The testis structure analyzed from the fetuses with prune belly syndrome showed lower concentration of Leydig cells. We did not observe differences in number and diameter of the seminiferous tubules and collagen distribution in prune belly syndrome.

## Figures and Tables

**Table 1:** The table shows the age and the fetal parameters of our sample: 7 male fetuses without anomalies and 3 male fetuses with prune belly syndrome. The 10 fetuses had abdominal testis. WPC = age in weeks postconception; g = grams; CRL = crown-rump length, and cm = centimeters.

Fetus	Age (WPC)	Anomaly	Weight (g)	CRL (cm)
1	17	Prune belly	240	18
2	23	Prune belly	1100	25
3	31	Prune belly	2150	43
4	21	None	900	20
5	35	None	2860	34
6	12	None	430	22
7	21	None	401	21
8	16	None	210	18
9	19	None	380	21
10	18	None	250	18
